# The Effect of the CO_3_^2- ^to Ca^2+ ^Ion activity ratio on calcite precipitation kinetics and Sr^2+ ^partitioning

**DOI:** 10.1186/1467-4866-13-1

**Published:** 2012-01-26

**Authors:** Tsigabu A Gebrehiwet, George D Redden, Yoshiko Fujita, Mikala S Beig, Robert W Smith

**Affiliations:** 1 University of Idaho-Idaho Falls Idaho Falls, 1776 Science Center Drive, Idaho Falls, ID 83402; 2 Idaho National Laboratory, P.O. Box 1625, MS 2208, Idaho Falls, ID 83415

## Abstract

**Background:**

A proposed strategy for immobilizing trace metals in the subsurface is to stimulate calcium carbonate precipitation and incorporate contaminants by co-precipitation. Such an approach will require injecting chemical amendments into the subsurface to generate supersaturated conditions that promote mineral precipitation. However, the formation of reactant mixing zones will create gradients in both the saturation state and ion activity ratios (i.e., $a_{C{O_{3}}^{2-}}/a_{Ca^{2+}}$). To better understand the effect of ion activity ratios on CaCO_3 _precipitation kinetics and Sr^2+ ^co-precipitation, experiments were conducted under constant composition conditions where the supersaturation state (Ω) for calcite was held constant at 9.4, but the ion activity ratio $\left(r=a_{C{O_{3}}^{2-}}/a_{Ca^{2+}}\right)$ was varied between 0.0032 and 4.15.

**Results:**

Calcite was the only phase observed, by XRD, at the end of the experiments. Precipitation rates increased from 41.3 ± 3.4 μmol m^-2 ^min^-1 ^at *r = *0.0315 to a maximum rate of 74.5 ± 4.8 μmol m^-2 ^min^-1 ^at *r = *0.306 followed by a decrease to 46.3 ± 9.6 μmol m^-2 ^min^-1 ^at *r *= 1.822. The trend was simulated using a simple mass transfer model for solute uptake at the calcite surface. However, precipitation rates at fixed saturation states also evolved with time. Precipitation rates accelerated for low *r *values but slowed for high *r *values. These trends may be related to changes in effective reactive surface area. The $a_{C{O_{3}}^{2-}}/a_{Ca^{2+}}$ ratios did not affect the distribution coefficient for Sr in calcite (D^P^_Sr_^2+^), apart from the indirect effect associated with the established positive correlation between D^P^_Sr_^2+ ^and calcite precipitation rate.

**Conclusion:**

At a constant supersaturation state (Ω = 9.4), varying the ion activity ratio affects the calcite precipitation rate. This behavior is not predicted by affinity-based rate models. Furthermore, at the highest ion ratio tested, no precipitation was observed, while at the lowest ion ratio precipitation occurred immediately and valid rate measurements could not be made. The maximum measured precipitation rate was 2-fold greater than the minima, and occurred at a carbonate to calcium ion activity ratio of 0.306. These findings have implications for predicting the progress and cost of remediation operations involving enhanced calcite precipitation where mineral precipitation rates, and the spatial/temporal distribution of those rates, can have significant impacts on the mobility of contaminants.

## Background

Engineering the precipitation of calcite in groundwater has been proposed as a means for remediation of the radionuclide strontium-90, a byproduct of uranium fission and a common contaminant at nuclear facilities in the U.S. and abroad [1-4]. Because of the relatively short half-life of ^90^Sr (29 yrs), and the compatibility of calcite within many subsurface environments, *in situ *immobilization of ^90^Sr by co-precipitation within calcite offers a potential long-term solution to the problem of ^90^Sr contamination. Co-precipitated ^90^Sr should be immobilized in environments where calcite is stable. After 300 years >99.9% of the radioactivity will be eliminated.

Strategies to manipulate calcite precipitation in the subsurface for the purpose of sequestering ^90^Sr are based on a well established understanding of the kinetics of low temperature calcite precipitation and dissolution [5-11] and of Sr^2+ ^partitioning into calcite [12-21]. However, predicting and controlling the rate and spatial distribution of calcium carbonate precipitation within a heterogeneous porous medium, such as the subsurface, remains technically challenging. The subsurface is an inherently poorly mixed system, and therefore attempts to manipulate mineral precipitation by injecting reactants (i.e., calcium, carbonate, or precursors) must confront the reality that chemical gradients will be formed. These gradients can result in chemical conditions that are far from equilibrium, unlike those addressed by the majority of the studies and reviews cited above. Such gradients can exist from the pore-scale to the macro-scale depending on the strategy used to introduce reactants. This means that in addition to variable saturation states with respect to CaCO_3 _phases, the ratio of reacting ions (calcium and carbonate) will vary spatially across the treatment zone and over time as the precipitation reaction propagates and removes the components from solution. This situation is made more complicated because the mineral product formed can fill pores and modify permeability, resulting in changing solute transport and mixing. Accurate predictions of reaction rates (and treatment progress) must take into account the impacts of these evolving gradients.

The rate of calcite precipitation is commonly described by an affinity-based rate law expressed by:

(1) $$R_{i}=k_{i}\cdot A_{T,i}\cdot {\left(\Omega^{n}-1\right)}^{m}$$

where Ω is the saturation state (Ω):

(2) $$\Omega =\frac{a_{CO_{3}}2{-}^{*}a_{Ca}2+}{K_{sp}}$$

*K_sp _*is the equilibrium solubility product of pure calcite, *R_i _*is the precipitation rate, *k_i _*is a rate constant, *A_T, i _*is the total surface area, and *m *and *n *are exponents. According to this model, the rate is a function only of the ion activity product, not the ion ratio. The thermodynamic basis for the affinity-based model is generally accepted to be valid under near equilibrium conditions. However, detailed studies at the solid-solution interface have revealed mechanistic differences in how individual ions (calcium vs. carbonate) are incorporated into specific locations (e.g., step kinks) on the mineral surface [22-25]. Other studies have reported macro-scale observations of the impact of ion ratios on calcite precipitation [20,26-28], which are described below.

Lin and Singer [27] investigated the effect of seed material and solution ion activity ratios at a constant saturation state (Ω = 5.3) on the kinetics and mechanisms of calcite precipitation. For a range of ratios of total carbonate to calcium, equivalent to ion activity ratios (${\text{a}}_{\text{C}{{\text{O}}_{\text{3}}}^{2-}}/{\text{a}}_{\text{C}{\text{a}}^{\text{2 + }}}$) ranging from 0.0002 to 1, Lin and Singer reported that the precipitation rate increased with an increasing ratio of total carbonate to calcium (C_T_/Ca^2+^). The maximum difference between the lowest (C_T_/Ca^2+ ^= 0.13; *r = *0.00016) and highest (C_T_/Ca^2+^=10; *r = *0.9997) observed rate was 3-fold.

Tai *et al*. [28] investigated the effect of the ion ratio on the rate of seeded calcite precipitation, using a fluidized-bed reactor. At Ω = 4 and pH 9.5, they reported that a maximum rate was observed at a ${\text{a}}_{\text{C}{{\text{O}}_{\text{3}}}^{2-}}/{\text{a}}_{\text{C}{\text{a}}^{\text{2 + }}}$ value of approximately 0.4; the maximum rate was approximately 35% greater than the lowest rate measured (at ${\text{a}}_{\text{C}{{\text{O}}_{\text{3}}}^{2-}}/{\text{a}}_{\text{C}{\text{a}}^{\text{2 + }}}$ = 0.083). Relative to the maximum, the calcite precipitation rate appeared to drop away less steeply with decreasing ${\text{a}}_{\text{C}{{\text{O}}_{\text{3}}}^{2-}}/{\text{a}}_{\text{C}{\text{a}}^{\text{2 + }}}$ compared to the other side, although the range of conditions tested was limited to $a_{C{O_{3}}^{2-}}/a_{Ca^{2+}}$ ratios smaller than 1.

More recently, Nehrke *et al*. [20] determined the growth rate of single calcite crystals (determined by change in mass) under conditions of constant supersaturation at Ω = 16 ± 2 and Ω = 5, and constant pH (10.2), but varying carbonate and calcium ion ratios in solution. They concluded that the precipitation rate was a maximum when the solution concentrations of calcium and carbonate ions were equal, and that the rate dropped in a symmetric manner on either side of the maximum. However, close examination of the reported data of Nehrke *et al*. [20] indicates that the maximum rate they measured occurred at *r *= 0.6. The difference between the highest rate (at ${\text{a}}_{\text{C}{{\text{O}}_{\text{3}}}^{2-}}/{\text{a}}_{\text{C}{\text{a}}^{\text{2 + }}}$ = 0.6) and the lowest rate (at ${\text{a}}_{\text{C}{{\text{O}}_{\text{3}}}^{2-}}/{\text{a}}_{\text{C}{\text{a}}^{\text{2 + }}}$ = 0.003 or 45) was much greater (~15 ×) than reported by Lin and Singer [27] and Tai *et al*. [28]. Nehrke *et al*. [20] also investigated the dependence of Sr^2+ ^partitioning between calcite and the parent solution on calcite growth kinetics and the ion ratio and reported that Sr partitioning increases with increasing rates of precipitation.

The aforementioned studies confirm that the solution stoichiometry of the reactant ions can indeed affect the rate of calcite precipitation. Both Nehrke *et al*. [20] and Tai *et al*. [28] reported observing rate maxima at particular stoichiometries although they differ with respect to both the ion ratio at which the maximum was observed and the magnitude of the effect (i.e., the range between the maximum and minimum rates). Whether the rates decrease symmetrically on either side of the maximum is not clear.

The objective of this study was to combine an investigation of the effect of the ion activity ratio (i.e., ${\text{a}}_{\text{C}{{\text{O}}_{\text{3}}}^{2-}}/{\text{a}}_{\text{C}{\text{a}}^{\text{2 + }}}$) on the calcite precipitation rate over a broad range of activity ratios with measurements of Sr^2+ ^partitioning during calcite precipitation. The ion activity ratio *r *(= ${\text{a}}_{\text{C}{{\text{O}}_{\text{3}}}^{2-}}/{\text{a}}_{\text{C}{\text{a}}^{\text{2 + }}}$) was varied over three orders of magnitude, from 0.0032 to 4.15. We conducted the study at pH 8.5, which is reasonably achievable in subsurface environments, and a degree of supersaturation (Ω = 9.4) that is plausible for engineered conditions. The experiments were conducted under constant composition conditions where the ion activities and pH conditions are held constant as CaCO_3 _precipitates. Typically, researchers using this method only report rates based on initial linear portions of the titrant volume vs. time plots [27-29]. In our study, we extended the duration of some experiments in order to observe whether the plots of our data remained linear. Changes in the slope of the curve (reflecting changing rates of precipitation) can indicate changes in precipitation mechanisms, and we were interested in whether the ion ratio affected transitions between mechanisms.

## Experimental

### Materials

All solutions were prepared by dissolving ACS reagent grade chemicals (J.T. Baker^® ^unless mentioned otherwise) in ultra pure water (≈18 MΩ-cm; Barnstead, Dubuque, IA). Calcium and strontium solutions were prepared using CaCl_2_·2H_2_O and SrCl_2_·2H_2_O. Carbonate solutions were prepared using NaHCO_3 _and KCl (Acros Chemicals). KCl was added to the carbonate solutions to set the ionic strength of the final mixed solutions at I ≈ 0.1. All solutions were filtered through 0.10 μm pore-size filters using a low vacuum system before initial pH measurement and refrigeration. Cation titrant solutions contained 0.5 M CaCl_2_·2H_2_O; some titrant solutions also contained either 0.0017 (7 experiments) or 0.017 (7 experiments) M SrCl_2_. The carbonate titrant solutions consisted of 0.45 M Na_2_CO_3 _+ 0.05 M NaHCO_3_. Two types of calcite seed were used: powder with a specific surface area of 1.29 m^2^/g (reported by manufacturer; J.T. Baker) and single Iceland spar crystals of typical dimension 10 mm × 6 mm × 5 mm (Ward's Scientific).

### Methods

Experiments were conducted using a constant composition technique adapted from previous studies [30,31]. Each experiment was conducted at 25°C, pH 8.5 ± 0.01, and Ω = IAP/*Ksp *= 9.4 ± 0.5 where IAP is the ion activity product and *Ksp *for pure calcite at 25°C = 10^-8.48 ^[8]. The carbonate to calcium ion activity ratio, *r *= ${\text{a}}_{\text{C}{{\text{O}}_{\text{3}}}^{2-}}/{\text{a}}_{\text{C}{\text{a}}^{\text{2 + }}}$, was set at values ranging from 0.0032 to 4.15. The solution compositions (speciation) for Ω = 9.4 ± 0.5 and Ionic strength (I) ≈ 0.1 were calculated using the SpecE8 module of Geochemist's Workbench™ (RockWare Inc, Golden, CO) with the thermodynamic database *thermo.dat *provided by Geochemist's Workbench™ (Described in more detail below). Thirty-three of the 74 individual experiments included Sr^2+ ^(0.1 mM) in the initial reactor solutions to test the effect of solution composition and precipitation rate on Sr partitioning. Four of the experiments included Sr^2+ ^in the initial reactor solutions at either 53 nM (3 experiments) or 5.3 μM (1 experiment) as well as Sr^2+ ^in the cation titrant solutions. None of the experiments that contained Sr^2+ ^in the titrant were included in the calculations of Sr distribution coefficients. Experimental conditions are summarized in Table 1.

**Table 1:** Experimental matrix, calculated CO_2 _fugacity, precipitation rate (R), curvature (*a*) of titrant volume vs. time plots (described in text), average mol % Sr and D^P^_Sr_^2^^+^.

	Reactor Solution composition					Results			
	
Experiment ID*	*r *(aCO_3_^2-^/aCa^2+^)	Ω	CaCl_2 _(M)	NaHCO_3 _(M)	log CO_2 _(g)	R _Ave _(μmol/m^2^/min) ± StdErr	a(Curvature)	Sr (mol %)	D^P^_Sr_^2+^**
IR-1(6)	0.0032	9.0	0.009	0.001	-3.85	60.6 ± 6.6	3.5E-04	0.14	0.086 ± 0.01(4)
IR-2(3)	0.0315	9.3	0.003	0.003	-3.34	41.3 ± 3.4	1.1E-04	-	--
IR-3(10)	0.0728	9.2	0.002	0.0045	-3.16	64.1 ± 3.9	8.2E-05	0.47	0.066 ± 0.01(4)
IR-4(8)	0.1120	10.0	0.0017	0.0058	-3.05	59.0 ± 1.1	3.1E-05	0.68	0.078 ± 0.01(4)
IR-5(4)	0.1528	10.4	0.0015	0.0069	-2.98	64.2 ± 1.3	3.1E-05	1.09	0.105 ± 0.01(4)
IR-6(10)	0.3062	8.9	0.001	0.009	-2.86	74.5 ± 4.8	9.8E-05	1.53	0.102 ± 0.02(4)
IR-7(7)	0.5336	9.4	0.0008	0.0122	-2.73	58.8 ± 2.7	1.5E-07	1.12	0.081 ± 0.01(4)
IR-8(8)	0.7312	9.0	0.00068	0.014	-2.67	50.8 ± 2.6	-3.8E-05	1.03	0.081 ± 0.01(4)
IR-9(8)	0.9624	8.9	0.0006	0.016	-2.61	62.3 ± 4.4	-3.1E-05	0.45	0.091 ± .001(3)
R-10(6)	1.8220	9.7	0.00048	0.023	-2.45	46.3 ± 9,6	-7.9E-05	0.12	0.053 ± .001(2)
IR-11(5)	4.1500	7.6	0.0003	0.031	-2.33	0.0	NA	-	--

Numbers in parentheses indicate *total number of replicates per experimental condition and **number of replicates used for strontium distribution coefficient calculations. The "--"sign is to indicate experiments where no Strontium was added. NA = Rate data not measured

Metastable solutions (500 ml volume) at Ω = 9.4 ± 0.5 were first introduced into a temperature-controlled (water-jacketed) stirred (at 150 RPM) 1 liter reactor by adding the same volume of separate chilled solutions of CaCl_2 _and NaHCO_3_. The mixture was brought to 25°C followed by pH adjustment to 8.5 using 0.05 M KOH. Metastability of the solution was inferred by a stable pH for at least 30 minutes. Approximately 100 mg calcite seed powder along with a single Iceland spar crystal (approximate size ~10 mm × 6 mm × 5 mm) were then added to initiate precipitation. The outlet of the reactor was located above the solution level to prevent particulate loss from the system.

Constant composition was maintained in the reactor through the use of an automated titration system; a decrease in solution pH due to calcium carbonate precipitation triggered the addition of solutions (at a rate of 10 μl/s) containing the cation and anion in equal amounts until the reactor pH returned to the target value of 8.5. The potential for spontaneous nucleation due to high, localized concentrations of reactants was minimized by locating the titrant entrances as far away from each other as possible, and stirring at 150 rpm (overhead propeller). The stirring also served to keep the seed materials in suspension. During the first hour of reaction, 2 ml sample volumes were collected from the reactor every 10-20 minutes for analysis of solution composition; in the longer experiments, samples were collected at 30 minute intervals during the second hour. In this system, the rate of titrant addition represents the rate of mineral precipitation. Typically, researchers using analogous methods only report on the initial linear portions of the titrant volume vs. time plots [27-29]; in our study, we extended the duration of some experiments in order to observe whether in fact the plots remained linear.

### Analytical Techniques

Inductively coupled plasma mass spectrometry (ICP-MS; Agilent 7500c) and ion chromatography (IC; Dionex) were used to measure the concentrations of cations (Ca^2+^, Sr^2+^, Na^+^, K^+^) and anions (Cl^-^) in the solution samples. A total organic carbon analyzer (Shimadzu TOC-VVSH) was used to measure total inorganic carbon (TIC). Samples for ICP-MS were acidified with nitric acid immediately after sampling whereas as those samples for Cl and TIC measurements were refrigerated with minimal headspace. Solid samples that had been filtered and oven dried for 15 minutes at 60°C at the end of the experiments were dissolved for calcium and strontium measurement by ICP-MS. ICP-MS, IC and TIC measurements were made using methods recommended by the instrument manufacturers. Oven dried precipitates from all experiments were rinsed with ethanol, oven dried, and examined using x-ray diffraction (XRD) and scanning electron microscopy (SEM). XRD was performed using a Bruker D8 Advance X-ray diffractometer (Bruker AXS, Congleton, Cheshire, UK) with Cu Kα_1/2 _emission using a Goebel mirror. The accelerating voltage was 40 KeV with a current of 30 mA and the step size was 0.02° with an integration time of 2 seconds. SEM was performed using an FEI Q650 FEG (Field Emission Gun) microscope in high vacuum mode with an accelerating voltage of 20 kV and an ETD (Everhartt-Thornley detector) for imaging.

### Calculation of precipitation rates

The precipitation rate can be expressed as the moles of calcite precipitated over time (based upon the volume of titrant added) normalized to the total surface area of the seed crystals, as shown in equation (3):

(3) $${\text{R}}_{\text{precipitation}}=\frac{\text{dv}}{\text{dt}}\frac{\text{c}}{\text{A}}$$

where dv/dt is the slope of the titrant volume versus time curve, c is the concentration of the titrant (0.5 M Ca^2+ ^and 0.5 M CO_3_^2-^), and A is the total surface area of the seed crystals (A (m^2^) = [Mass Seed Crystals added (g)] * [Surface Area (m^2^/g)]).

A linear relationship between titrant volume and time can be indicative of no significant changes in surface area [27,29]. However, we found long-term deviations from linearity in our experiments (discussed in the results section). Hence, calculations of dv/dt are based on data from the first 20 minutes of the experiments where departure from linearity was minimal.

### Determination of the Sr^2+^distribution coefficient

In suspensions maintained at constant composition by replenishing components lost during precipitation, the distribution coefficient of Sr^2+ ^or other co-precipitating metals between the calcite and the parent solution are determined by the Henderson-Karcek distribution coefficient equation [12]:

(4) $${{\text{D}}^{\text{P}}}_{\text{S}{\text{r}}^{2+}}=\frac{{{\text{X}}^{\text{P}}}_{\text{S}{\text{r}}^{\text{2 + }}}/{{\text{X}}^{\text{P}}}_{\text{C}{\text{a}}^{\text{2 + }}}}{{{\text{M}}^{\text{S}}}_{\text{S}{\text{r}}^{\text{2 + }}}/{{\text{M}}^{\text{S}}}_{\text{C}{\text{a}}^{\text{2 + }}}}$$

where X^P^_Sr_^2+ ^and X^P^_Ca_^2+ ^are the mole fractions of Sr^2+ ^and Ca^2+ ^in the precipitates (superscript P) and M^S^_Sr_^2+ ^and M^S^_Ca_^2+ ^are the molar concentrations in the solution (superscript S). Because experiments that include Sr in the replenishment titrant result in a continual increase in the solution Sr/Ca ratio, only experiments in which Sr was not replenished by the titrant are used to determine strontium distribution coefficients.

### Speciation calculations

Ion activities for Ca^2+ ^and CO_3_^2- ^were calculated for each experimental condition using solution compositions and The Geochemist Workbench GWB Essentials release 8.0 [32]. The thermodynamic database provided with Geochemist Workbench, *thermo.dat*, was used for the calculation. Formation of solid phases was suppressed in the calculations since solutions poised for precipitation were metastable. The *thermo.dat *database was compiled by the geochemistry modeling group at Lawrence Livermore National Laboratories [33] and is based in large part on the SUPCRT [34] data compilation [32]. A copy of *thermo.dat *is available at http://www.gwb.com/thermo.htm

The ion activities for Ca^2+ ^and CO_3_^2- ^were used to calculate both ion activity ratios (*r *= $a_{C{O_{3}}^{2-}}/a_{Ca^{2+}}$) and ion activity products ($a_{C{O_{3}}^{2-}}/a_{Ca^{2+}}$) for calcite. Values of Ω were calculated from the ion activity products and the *K_sp _*for calcite of 10^-8.48 ^reported by Plummer and Busenberg [8], which was determined directly from solubility measurements rather than the values in *thermo.dat *that were calculated from thermodynamic properties of the individual ions and minerals using SUPCRT [34].

## Results

### Calcite precipitation rates and ion ratios

**Figure 1:**
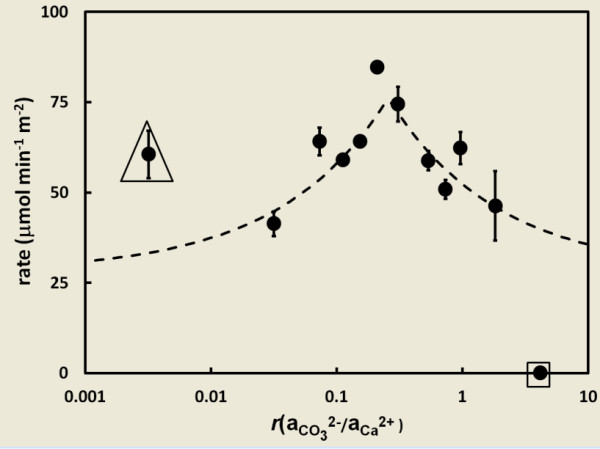
**Graphical Abstract: At a given supersaturation state, the conventional affinity-based rate law predicts a rate that is independent of the ion ratio (green horizontal line)**. However, the ion activity ratio influences the instantaneous rate, changes in the rate over time, and the nature of precipitation kinetics at extreme ion ratios. Calcite precipitation rate as a function of ion activity ratio at constant composition and supersaturation (Ω = 9.4 ± 0.5). Error bars represent the standard error. The dashed line represents a simple kinetic model of the experimental data, discussed in the text.

The experimental conditions and calculated rate and Sr distribution coefficient data are summarized in Table 1. Figure 1 shows the relationship between calcite precipitation rates and the ion activity ratio. The difference between the minimum and maximum measured rates across the range of ion ratio values tested (from *r *= 0.0032 to *r *= 4.15) is approximately 2-fold, with a maximum rate (74.5 ± 4.8 μmol m^-2 ^min^-1^) observed at *r *= 0.306 (Figure 1). The absence or presence of Sr^2+ ^at up to 0.1 mM in the reactor solution and up to 0.017 M in the titrant solution appeared to have no effect on the rate of calcite precipitation that could be statistically distinguished from the standard deviations calculated for the replicate experiments. Hence, in calculating the rates for any one carbonate to calcium ion activity ratio all experiments are treated as replicates regardless of the presence or absence of Sr^2+ ^in the reactor and titrant solutions.

Kinetics at the two "extremes" of the range of tested ion ratios exhibited different behavior. These behaviors were confirmed by running at least five replicate experiments for each of these two conditions. At the lowest ion activity ratio tested (${\text{a}}_{\text{C}{{\text{O}}_{\text{3}}}^{2-}}/{\text{a}}_{\text{C}{\text{a}}^{\text{2 + }}}$ = 0.0032; IR-1 in Table 1 and marked by the triangle in Figure 1), a metastable solution was not achievable. Precipitation commenced immediately upon mixing of the calcium and carbonate solutions. The titrant volume vs. time curves for these experiments showed an immediate exponential increase in the apparent precipitation rate. At the high end of the range of ion activity ratios (${\text{a}}_{\text{C}{{\text{O}}_{\text{3}}}^{2-}}/{\text{a}}_{\text{C}{\text{a}}^{\text{2 + }}}$ = 4.15; IR-11 in Table 1 and marked by square in Figure 1) no evidence for precipitation was observed after addition of seed crystals. Further addition of seed material did not stimulate detectable precipitation. These two extreme data points are not used in the modeling of experimental data (discussed in a subsequent section).

**Figure 2:**
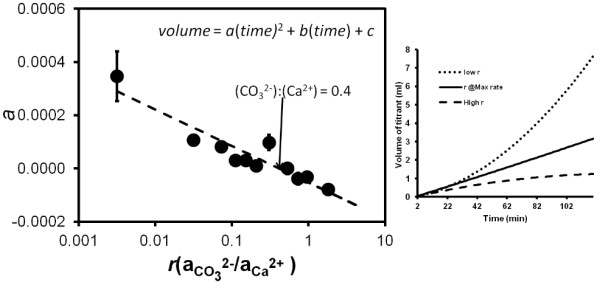
**Graphical Abstract: At a given supersaturation state, the conventional affinity-based rate law predicts a rate that is independent of the ion ratio (green horizontal line)**. However, the ion activity ratio influences the instantaneous rate, changes in the rate over time, and the nature of precipitation kinetics at extreme ion ratios. The observed curvature (*a*) for the titrant volume vs. time plots for different ion activity ratio conditions. Each point represents the average curvature for replicate experiments at a given ion ratio. The inset shows examples of individual titrant volume vs. time curves.

As noted in the methods section, we observed that the rates did not necessarily remain constant beyond approximately 20 minutes. Titrant volume vs. time curves tended to curve upward or downward with time. An interesting observation is that the direction of curvature appeared to be related to the ion activity ratio: a positive deviation (upward curve, indicating increasing rate with time or amount of calcite precipitated) is associated with lower *r *values and a negative deviation (downward curve, indicating decreasing rate) is associated with higher *r *values. Figure 2 shows the observed curvature a (the coefficient of the squared term when the titrant volume vs. time curve is fit with a quadratic equation) plotted vs. the ion activity ratio. Each point represents the average curvature for replicate experiments at a given ion ratio. The inset shows examples of the curvature of individual titrant volume vs. time curves. The linear trend in the data indicates that the transition from an upward to a downward deviation occurred near the ion ratio where the maximum precipitation rate was observed, ${\text{a}}_{\text{C}{{\text{O}}_{\text{3}}}^{2-}}/{\text{a}}_{\text{C}{\text{a}}^{\text{2 + }}}$ = 0.306.

### Characterization of solids

**Figure 3:**
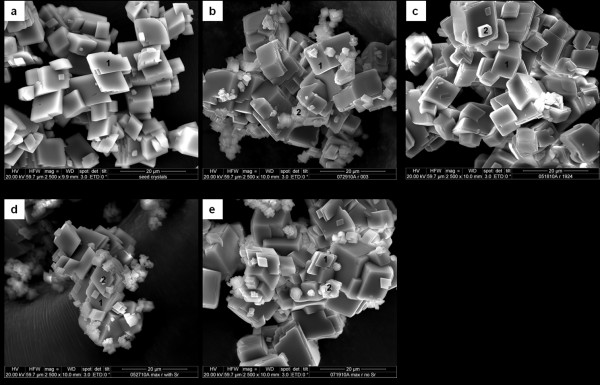
**Graphical Abstract: At a given supersaturation state, the conventional affinity-based rate law predicts a rate that is independent of the ion ratio (green horizontal line)**. However, the ion activity ratio influences the instantaneous rate, changes in the rate over time, and the nature of precipitation kinetics at extreme ion ratios. SEM Images of (a) unreacted seed materials; and of newly formed calcite crystals on seed materials under conditions (b) *r *= 0.0032 with 0.1 mM Sr^2+^; (c) r = 1.822 with 0.1 mM Sr^2+^; (d) *r *= 0.306 and 0.1 mM Sr^2+^; and (e) *r *= 0.306 with no Sr^2+^. Numbers indicate beam location for EDS qualitative elemental mapping.

SEM images of the final calcite products from experiments are shown for the two extreme ion ratio conditions (Figures 3b, c) and for the maximum growth rate condition at *r *= 0.306 (Figures 3d, e). The original seed material is shown in Figure 3a. Samples from the lowest *r *value (*r *= 0.0032), where a metastable solution could not be prepared, showed clumps of small irregular crystallites that appeared to be distributed randomly between crystal faces and edges (Figure 3b). The indiscriminate distribution of the crystallites could be due to surface nucleation or attachment of small crystals formed by homogeneous nucleation. In each of the replicate experiments (6 total) conducted at *r *= 0.0032 spontaneous nucleation occurred upon mixing of the calcium and carbonate solutions. In the high *r *experiment (*r *= 1.822; Figure 3c) small attached crystallites are rarer, and the edges of the crystals are more consistent with calcite growth along steps. The images in Figure 3d and 3e (*r *= 0.306) are similar to that for *r *= 0.0032. No visible differences are observed in the presence (Figure 3d) or absence (Figure 3e) of Sr^2+^, consistent with the observations with respect to the effect of Sr^2+ ^on precipitation rates. Where Sr is present in the initial solution, EDS mapping detected Sr in the small crystallites on top of the seed crystals. XRD analyses were consistent with the formation of only calcite (i.e., neither aragonite nor strontianite was detected), regardless of the presence or absence of Sr (data not shown).

### Sr^2+ ^partitioning

**Figure 4:**
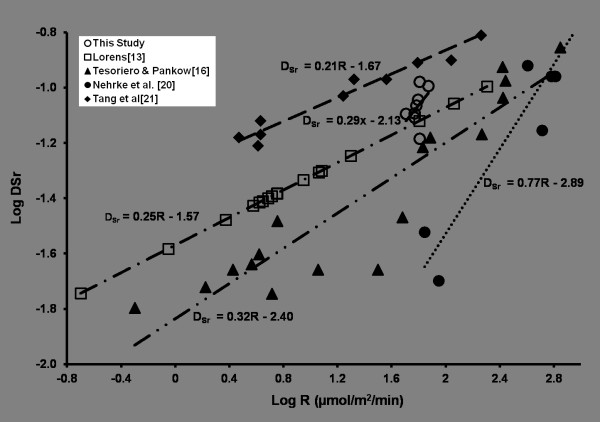
**Graphical Abstract: At a given supersaturation state, the conventional affinity-based rate law predicts a rate that is independent of the ion ratio (green horizontal line)**. However, the ion activity ratio influences the instantaneous rate, changes in the rate over time, and the nature of precipitation kinetics at extreme ion ratios. Log of strontium distribution coefficient as a function of calcium carbonate precipitation rate; our study compared to other similar previous studies (Lorens [13]; Tesoriero & Pankow [16]; Nehrke *et al*. [20]; and Tang *et al*. [21]). Lines shown for each respective data set represent linear fits of the data. D_Sr _Data from Lorens [13] are calculated based on the best fit line equation (LogD_Sr _= 0.249*LogR-1.57) of the experimental data used.

**Figure 5:**
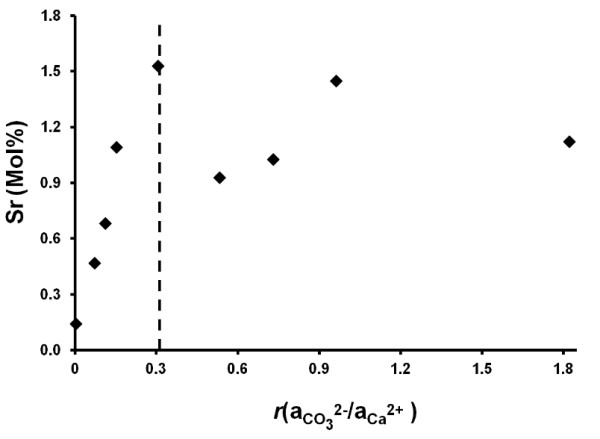
**Graphical Abstract: At a given supersaturation state, the conventional affinity-based rate law predicts a rate that is independent of the ion ratio (green horizontal line)**. However, the ion activity ratio influences the instantaneous rate, changes in the rate over time, and the nature of precipitation kinetics at extreme ion ratios. Strontium mole fraction plotted vs. ion activity ratio *r*. The vertical line indicates the ion activity ratio at the highest observed precipitation rate.

Calculated D^P^_Sr_^2+ ^values varied between 0.053 and 0.105. These values are within the ranges reported by others for Sr partitioning during abiotic calcite precipitation; Figure 4 shows our distribution coefficients (open circles) plotted against the corresponding calcite precipitation rate, along with data reported in the literature from other studies. Compared to the other studies, only a narrow range of precipitation rates was generated in our study. Nevertheless, our data are consistent with the findings of others [13,14,16,20] that strontium partitioning into calcite increases with the calcite precipitation rate. The Sr mol % is plotted against the ion activity ratio (*r*) in Figure 5. A vertical line is drawn at *r = *0.306 where the rate of precipitation was a maximum.

## Discussion

We observed a maximum calcite precipitation rate at *r *= 0.306, which is similar to the findings of Tai *et al*. [28] who reported a maximum rate at $a_{C{O_{3}}^{2-}}/a_{Ca^{2+}}$ = 0.36. This result is at odds with the stated contention of Nehrke *et al*. [20] that claimed the maximum rate should occur at the stoichiometric ion ratio for calcite of one. However, as noted previously, the maximum precipitation rate reported by Nehrke *et al*. [20] actually occurred at *r *= 0.6. A maximum growth rate at *r *=1 was also reported by Perdikouri *et al*. [23] in a study where the rate of precipitation was estimated by measuring the rate of etch pit closure and monolayer step growth for the {104} plane of cleaved Icelandic Spar. In the Perdikouri *et al*. [23] study, the degree of asymmetry in the precipitation rate around the maximum was more pronounced than what we observed. We do not attempt to resolve the differences in findings in this case since our approach measured behavior averaged over all surfaces rather than the isolated behavior of specific planes of calcite; however, the complexity of the precipitation process is evident.

Chernov *et al*. [35] developed a model for non-Kossel crystal growth that incorporates frequency factors for the attachment of individual ions at crystal growth steps. The model assumes that the frequency factors are independent of ion type (therefore, a single frequency factor is applied). Under this assumption, the model predicts a decrease in precipitation rate that is symmetric around a maximum rate at an ion ratio equal to the stoichiometric ratio of solid phase (1 for calcium carbonate). The prediction of symmetry, and the assumption of equal attachment frequency factors, was supported by atomic force microscopy measurements of step growth rates on calcium and magnesium oxalates. Equivalent attachment frequency factors may be valid in some cases but additional processes affecting the frequencies of attachment are likely, such as deprotonation steps for bicarbonate ions, dehydration of cations or unequal transport of constituent ions to growth sites in mixed electrolyte systems. The significance of each process will obviously depend on the solid phase and solution composition, which includes background electrolytes. The occurrence of both precipitation rate maxima at ion ratios different from the stoichiometric ratio, and asymmetry in the dependence of rate on ion ratio are observed, such as in a study of barium sulfate growth by Kowacz *et al*. [36]. Table 2 summarizes conditions and results for studies of calcium carbonate precipitation along with results from the current study.

**Table 2:** Summary of calcite precipitation rates (R) as a function of ion activity ratio (aCO_3_^2^^-^/aCa^2^^+^) in this and previous studies

Ranges of aCO_3_^2-^/aCa^2+^	Ranges of R (mol m^-2 ^min^-1^)	Ω (calc)	pH	Ionic Strength(M)	Electrolyte	References
0.048 - 39.34	3.84E-06 - 2.18E-05*	5.4 - 7.4	6.97 - 7.55	0.1 - 2.0	NaCl	Zhang&Dawe [26]
0.0002- 0.9997	5.17E-05 - 1.46E-04^± ^	5.3	7.21 - 9.01	0.1	KCl	Lin&Singer [27]
0.056 - 1.00	1.71E-04 - 2.21E-04^‡^	1	8.50	0.018	NaCl	Tai *et al*.[28]
0.003 - 57	5.80E-05 - 8.87E-04**	5 - 19	10.2	0.1	NaCl	Nehrke *et al*. [20]
0.052 - 0.194	2.96E-06 - 2.7E-04^± ± ^	4.5 - 17.0	7.6 - 9.4	0.036	NH_4_Cl	Tang *et al*. [21]
0.003 - 1.82	4.13E-05 - 8.47E-05	8.9 - 10.4	8.500	0.1	KCl	Current Study

*Values calculated based on data and equations given in table 1 of Zhang & Dawe [26]; ^± ^rate values used for calculation are estimated from Figure 6 in Lin & Singer [27]; ^‡^rate values used for calculation are estimated from Figure 8 in Tai *et al*.[28], ** Values calculated based on data given in table 3 of Nehrke *et al*. [20], ^± ± ^Values calculated based on data and equations given in table 1 of Tang *et al*. [21]

Regardless of where along the range of solution composition the calcite precipitation rate maximum occurs, it is apparent that the precipitation rate depends on the ion ratio. Furthermore, the dependence of precipitation rates on ion transport across the solid-solution boundary should also be a function of the ion ratio. To use an extreme example, if only a single ion of a species (i.e., calcium or carbonate) is present and ions of the other species make up the remainder of the ion product, the precipitation of calcite will obviously be limited by transport of the minority component, if not reversed (dissolution) due to the concentration gradient between the bulk solution and the solid-solution interface. The effect may also be consistent with observations of Stack and Grantham [25] where at high ion ratios etch pits were observed on cleavage planes under supersaturated conditions. Such mass transport control of the precipitation rate mechanism has been quantitatively described in previous studies [37-39]. Assuming a transport or surface controlled precipitation mechanism can be approximated by a simple linear relationship, we can develop a tractable model that illustrates the effect of ion ratios on precipitation rates. In order for calcite to grow both CO_3_^2- ^and Ca^2+ ^ions must be brought to the surface. This can be accomplished by transport and attachment of an aqueous CaCO_3 _ion pair, where the concentration of the ion pair in solution can be calculated using equilibrium assumptions and speciation calculations. The rate (R_1_) is given by a first-order rate law with rate constant of k_1_:

(5) $${\text{R}}_{1}={\text{k}}_{1}\left[\text{CaC}{{\text{O}}_{3}}^{0}\right]$$

At a constant value of Ω, [CaCO_3_^0^] is constant (since the ion activity product is constant) and R_1 _is therefore independent of the ion ratio. However, the calcite precipitation rate can also be regulated by the rate of transport and attachment of individual CO_3_^2- ^and Ca^2+ ^ions. The rate of transport or attachment for the individual ions is given by:

(6) $${\text{R}}_{2}={\text{k}}_{2}\left[\text{C}{\text{a}}^{2+}\right]$$

(7) $${\text{R}}_{3}={\text{k}}_{3}\left[\text{C}{{\text{O}}_{\text{3}}}^{2-}\right]$$

Because the growth of calcite in the above model requires that both CO_3_^2- ^and Ca^2+ ^ion be added to the surface, the rate of calcite growth due to this simple model will be determined by the *slower *of R_2 _and R_3_. The overall rate of precipitation will be the sum of R_1 _and the slower of R_2 _or R_3_:

(8) $$\begin{array}{ll} \text{Rate} & =\text{min}\left\{{\text{R}}_{2},{\text{R}}_{3}\right\}+{\text{R}}_{1}\;\\ & =\text{min}\left\{{\text{k}}_{2}\left[\text{C}{\text{a}}^{2+}\right],{\text{k}}_{3}\left[\text{C}{{\text{O}}_{3}}^{2-}\right]\right\}+{\text{R}}_{1}\;\\ & \; \end{array}$$

Equations 6 and 7 can also be cast as diffusion rates where k_2 _and k_3 _are single ion diffusion coefficients and the ion concentration term is replaced by a concentration gradient expression which is the difference between the bulk solution ion concentration and the solid-solution interface concentration across a diffusion layer of constant thickness, e.g.,

(9) $$\begin{array}{ll} {\text{R}}_{2} & ={\text{D}}_{2}\left\{{\left[\text{C}{\text{a}}^{2+}\right]}_{\text{solution}}-\left[\text{C}{\text{a}}^{2+}\right]\text{interface}\right\}/\left\{\text{D}.\text{L}.\right\}\;\\ & =-{\text{D}}_{2}{\left[\text{C}{\text{a}}^{2+}\right]}_{\text{interface}}/\left\{\text{D}.\text{L}.\right\}+{{\text{C}}_{\text{Ca}}}^{2+}\;\\ & \; \end{array}$$

where D.L. is the diffusion layer thickness and the constant term C_Ca2+ _= D_2_{[Ca^2+^]_solution_}/{D.L.}. If the solid-solution interface concentration is near equilibrium because of rapid surface attachment of the ions, then R_2 _collapses to the constant term and equations 6 and 7 are the same as shown. This is because the drawdown in driving force (in a transport-limited environment) pulls the supersaturation state near equilibrium, which is the limit of solubility. If the solid-solution interface concentration is near equilibrium with the solid phase or at steady state, then both equations 6 and 7 would include the addition of constants. However, this does not affect the analysis.

The important implication of this conceptual model is that at high CO_3_^2-^:Ca^2+ ^ratios the addition of Ca^2+ ^will be the rate limiting growth step. At low ion activity ratios the addition of CO_3_^2- ^will be the rate limiting growth step. It is important to note that for a constant value of Ω, the ion activity product of (CO_3_^2-^)(Ca^2+^) will be constant. The concentrations of CO_3_^2- ^and Ca^2+ ^can vary, but not independently. The simulation of precipitation rate as a function of ion ratio according to the above conceptual model is presented as a dashed line in Figure 1. The values of R_1_, k_2 _and k_3 _(R_1 _= 4.0E-5 mol m^-2 ^min^-1^, k_2 _= 0.1 min^-1^, k_3 _= 0.33 min^-1^, Ω = 9.4) were determined by least squares fits to the data collected in this study, excluding the "extremes" (*r *= 0.0032 and *r *= 4.15; the data collected for those conditions are however shown in the figure, marked by triangle and square symbols). The lower value of k_2 _relative to k_3_, which is reflected in the occurrence of the maximum rate to the left of $a_{C{O_{3}}^{2-}}/a_{Ca^{2+}}$ = 1, is consistent with the suggestion by previous studies [12,36,39,40] that cation dehydration can be the rate limiting step for mineral precipitation. Deviations of the maximum precipitation rate from a stoichiometric ion ratio of one (for calcite) can be further influenced by unequal single ion diffusion coefficients, which cannot be calculated for most mixed electrolyte systems.

In measuring the rates of calcium carbonate precipitation under conditions of constant Ca^2+ ^and CO_3_^2- ^activities the assumption is that the total solid surface area in the reactor does not change appreciably and that calcium and carbonate are added at constant rates while maintaining a target pH of 8.5. Kazmierczak *et al*. [29] reported that a surface area increment of >15% results in deviation from linearity for plots of volume of titrant added as a function of time. The change in surface area was not measured independently in our study. However, a high-end estimate of the change in surface area can be approximated by assuming the seed crystals were individual cubes with smooth faces and that precipitation occurred as uniform epitaxial growth on all faces. Using a density of 2.71 g/cm^3 ^for calcite, the increase in surface area can be as high as 40% by the conclusion of a typical experiment. The increase in surface area will be less for epitaxial growth on most other geometries (precipitation in concave corners and edges will reduce surface area), but positive deviations in rates due to increased surface area are generally expected. However, as shown in Figure 2, long term changes in the rates of precipitation were not uniform. The rates of precipitation are observed to increase over time for *r *< 0.4, decrease for *r *> 0.4 and nearly constant for *r *~ 0.4.

Negative deviations from a constant precipitation rate are not consistent with increased surface area. One alternate explanation is that changes in ionic strength during an experiment resulted in changes in the individual activities of Ca^2+ ^and CO_3_^2- ^and therefore a change in the driving force for precipitation, in accordance with an affinity based model for precipitation kinetics. However, changes in ionic strength were less than 1% in our study, and thus not likely to significantly influence the ion activities in the experiments. In a previous study by Zuddas and Mucci [41] ionic strength (I = 0.1 - 0.93 M) was found to have little effect on the rate of calcite precipitation. Stephenson *et al*. [42] also reported that ionic strength variations had no measurable effect on the calcite precipitation rate. Although the effect of ionic strength should have been minor, the elemental or molecular composition of the electrolyte can be a complicating factor. Zhang and Dawe [26], for studies where the background electrolyte was NaCl, reported a 5-fold increase in calcite growth rate for an increase in ionic strength from 0.1 M to 2.0 M. Other studies [28,43,44], where the electrolyte was NaCl, observed similar variations in precipitation rates as functions of ion ratios as in our experiments (maximum precipitation rates at *r *= 0.3 to 0.4), but the overall rates of precipitation were significantly slower than what we observed in our experiment where KCl was the background electrolyte. Tai *et al*. [28] did report that calcite growth rates tripled over the range of ionic strengths from 0.002 to 0.0157 although in their experiments the NaCl concentrations were uncontrolled products of CaCl_2 _and Na_2_CO_3 _additions to a fluidized reactor. In each of these cases, increases in "ionic strength" resulted in either no or positive effects on precipitation rates rather than decreases. Nevertheless, these studies show that it can be important to distinguish between the effect of ionic strength on solute activity from the effects that individual electrolytes can have on the rates of solute incorporation into the solid matrix.

Another possible factor is particle aggregation or agglomeration. Aggregation can reduce the specific surface area by eliminating surfaces where particles grow together or can result in hindered transport of solutes to surfaces in the interior of the aggregates. SEM images for high *r *conditions (e.g., Figure 3c) show less evidence for surface nucleation with growth of smaller, high specific surface area crystals as compared to the images for the low *r *conditions (Figure 3b). However, whether aggregation was important under the high *r *conditions cannot be ascertained from the available data.

In considering the rate data reported in Table 1 and Figure 1, it is important to remember that the reported values reflect the net rates of precipitation and an extrapolation to the initial times in order to minimize artifacts caused by changes in surface area or the state of the seed crystals. Details of the underlying mechanisms for mineral precipitation are becoming increasingly refined from studies using atomic-scale microscopy [24,25,35,45,46]. It is apparent that net rates of precipitation reactions are an integration of transport, solute speciation, precipitation mechanisms that are unique to specific crystal planes, etc. It has been pointed out by Larsen *et al*. [24] and others that, in addition to the solution composition, other factors that contribute to net calcite growth rates include kink energies and geometries, ion dehydration, and the identity and quantity of reactive surface species. Our study is applicable for the improvement of field-scale models used to predict the distribution of mineral precipitates under engineered conditions where solute activity gradients can affect local rates of reaction.

As noted previously and shown in Figure 4, the Sr distribution coefficient results from our study, while limited, are consistent with previous work by others. The observed trend (increasing D_Sr _with log R) in our data is similar to what has been reported before [13,16,20,21]. Overall, the data do not indicate any independent effect of the solution composition on Sr partitioning, apart from the effect induced by greater calcite precipitation rates.

Another interesting observation is the relationship between the Sr mol % in the solid and ion activity ratio (*r*) shown in Figure 5. This relationship shows that the maximum Sr mol % corresponds to the maximum precipitation rate (marked by the vertical dash line at *r *= 0.306), however the behavior above and below this point is not symmetric. There is a clear positive linear trend for *r *< 0.306, but the trend for *r *> 0.306 has a less positive slope. This suggests that strontium incorporation could be a strong function of the prevalence of individual step growth rates or step properties in addition to changes in solute speciation.

## Conclusions

Under the conditions of our study (constant pH 8.5, Ω = 9.4 ± 0.5, and a range of ion activity ratios extending over 3 orders of magnitude) measured calcite precipitation rates ranged between 41.3 and 74.5 μmol m^-2 ^min^-1^. We observed a maximum rate at a non-equimolar ion activity ratio, specifically where the calcium activity is greater than that of carbonate (*r *= $a_{C{O_{3}}^{2-}}/a_{Ca^{2+}}$ = 0.306). The rate decreased symmetrically on either side of the maximum. Strontium partitioning into calcite appeared to increase with an increasing rate of calcite precipitation, consistent with other published studies. However, we did not observe that the ion ratio in itself affected Sr partitioning aside from the indirect effect associated with the positive correlation between D^P^_Sr_^2+ ^and calcite precipitation rate.

At one end of the ion activity ratio (*r *= $a_{C{O_{3}}^{2-}}/a_{Ca^{2+}}$ = 0.0032) continuum we observed rapid homogeneous precipitation and on the other end (*r *= $a_{C{O_{3}}^{2-}}/a_{Ca^{2+}}$ = 0.415) long term metastability of the reactant mixture. A similarly wide range of conditions may be expected to exist when reactants are introduced into the subsurface, with significant consequent effects on the temporal and spatial progress of the desired calcite precipitation reactions. This in turn will affect the risk and cost of remediation efforts. Additional research to examine these phenomena in detail within porous media is currently underway.

## Competing interests

The authors declare that they have no competing interests.

## Authors' contributions

MB initiated the setup of experiments and ran the initial experiments. TG conducted most of the experiments and data analysis. YF helped to draft the outline of the manuscript and together with TG provided most of the text. GR assisted in the design of the experiments, and contributed to the discussion and manuscript outline. RS assisted in analysis and interpretation of the data, constructed the kinetic model and contributed to the discussion.
